# Application of exposure enhancement technique combined with femoral condyle pushing technique in repairing the posterior horn of the medial meniscus under knee arthroscopy

**DOI:** 10.12669/pjms.38.6.5176

**Published:** 2022

**Authors:** Xinwei Liu, Dulei Xiang, Ying Zi, Tianyu Han, Chenchen Xue

**Affiliations:** 1 Xinwei Liu, Department of Orthopaedics, The General Hospital of Northern Theater Command, Shenyang 110016, People’s Republic of China; 2 Dulei Xiang, The General Hospital of North Theater Command, Training Base of Jinzhou Medical University Graduate, Shenyang 110016, People’s Republic of China; 3 Ying Zi, Department of Emergency Medicine, Graduate Training Base of Jinzhou Medical University, Air Force hospital of the northern theater of Chinese PLA, Shenyang, Liaoning,110042, People’s Republic of China; 4 Tianyu Han, Department of Orthopaedics, The General Hospital of Northern Theater Command, Shenyang 110016, People’s Republic of China; 5 Chenchen Xue, Department of Joint Surgery, Changhai Hospital, Navy Medical University, Shanghai, 200433, People’s Republic of China

**Keywords:** The posterior horn of the medial meniscus, Arthroscopy, Repair, Exposure enhancement technique, Femoral condyle pushing technique

## Abstract

**Objectives:**

To investigate the clinical efficacy of exposure enhancement technique and femoral condyle pushing technique applying in the posterior horn of the medial meniscus of the knee.

**Methods:**

From January 2016 to June 2019, 52 patients with injury in the medial meniscus treated in our department were selected. The horizontal tear of the posterior horn of the medial meniscus was repaired by exposure enhancement technique and femoral condyle pushing technique using the meniscus suture system. Postoperatively, the efficacy was evaluated using the Lysholm scoring system.

**Results:**

These 52 patients were all followed up for 3~18 months, with an average of 12.5 ± 7.3 months. The pain and activity of all patients were significantly improved compared with those before surgery.

**Conclusion:**

Exposure enhancement technique and femoral condyle pushing technique in the repair of the posterior horn of the medial meniscus presents satisfactory efficacy. It can improve the pain and activity of the knee, and enhance the stability of residual meniscus. Therefore, it is worth promoting.

## INTRODUCTION

Meniscal injury is a common knee joint injury in military and sports training, always caused by rotation of the knee joint in flexion and compression of the meniscus by the femoral condyle. It is easy to result in symptoms such as knee joint pain, genual asthenia and locking, which seriously reduces sports ability and personal quality of life.^1^-^3^ As an important structure of the knee, the meniscus not only plays a role of buffer and shock absorption, but also has an important contribution to the stability of the knee.^4^-^5^ Avulsion of the posterior horn of the medial meniscus can be caused by acute trauma or chronic degeneration, resulting in meniscal compression, loss of articular cartilage, osteophyte formation, and narrowing of the medial joint space. Over time, this can lead to symptomatic knee osteoarthritis. Although non-surgical treatment can alleviate symptoms, it is unlikely to alter the natural course of meniscus loss or the fate of the medial ventricle.^6^ It is often accompanied by quadriceps atrophy and even joint stiffness.

For these patients, arthroscopic surgery should be performed if necessary.^7^ Surgical repair of posterior angular meniscus avulsion can restore the anatomical and biomechanical functions of the meniscus and slow down or prevent degenerative joint diseases.^8^ Fifty-two patients who underwent arthroscopic exposure enhancement combined with femoral condyle pushing to repair the posterior corner of the medial meniscus were studied. Our objective was to evaluate the efficacy of exposure enhancement combined with femoral condyle pushing technique in meniscus repair.

## METHODS

A case summary of retrospective study was used in this study. From January 2016 to June 2019, 52 patients with injury in the medial meniscus treated in our department were selected by random number table method. All the 52 patients (52 knees) were male, aging 35-55 years (average age, 45.5 ± 8.7 years). The time of symptom onset was 1-12 weeks after injury, with an average of 5.5 ± 3.5 weeks.

###  Inclusion Criteria:

• The patients were all caused by sprain of the knee joint during military and sports training, and suffered from obvious pain when the affected knee squatted.
• Physical examination showed that knee hyperflexion test and medial joint line tenderness were both positive.
• Patients with poor response to conservative treatment.
• The injury of the medial meniscus of the knee (grade 3) was clearly diagnosed by MRI.
• Male patients.
• Patients with good joint stability.

### Exclusion Criteria:

• Patients with anterior and posterior cruciate ligament injury.
• Patients with organic diseases such as heart, brain and kidney.
• Patients who could not complete the follow-up.
• Patients who cannot independently sign informed consent forms.

### Ethical Approval

The study was approved by the Institutional Ethics Committee of the General Hospital of Northern Theater Command on September 20, 2019 (No. [2019]087), and written informed consent was obtained from all participants.

### Surgical method

**Fig.1:**
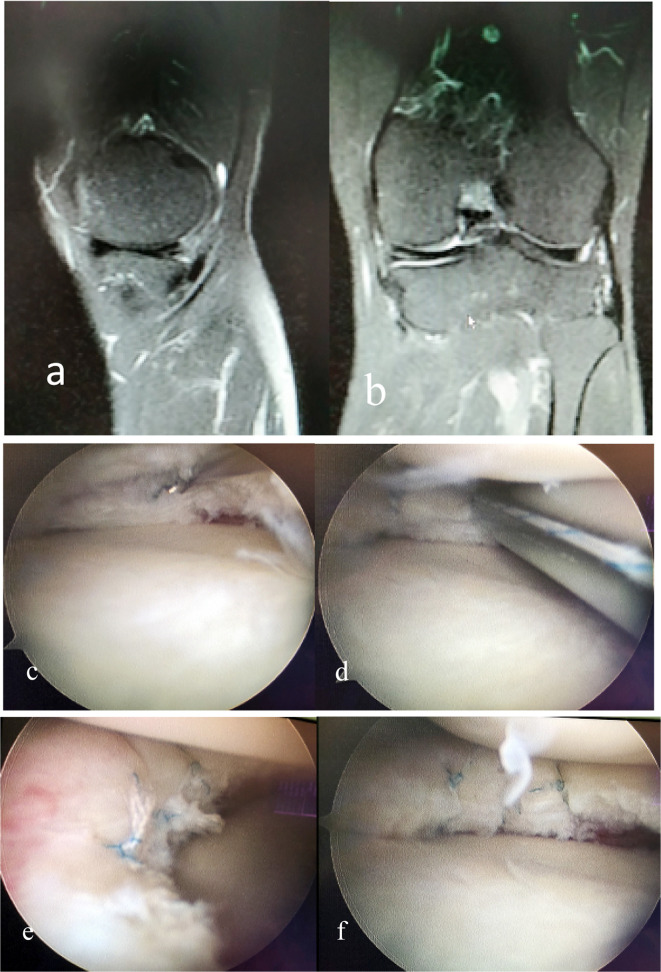
Male, 55 years old, injury of the posterior horn of the medial meniscus of the right knee. a, b: A horizontal tear of the posterior horn of the medial meniscus; c: the end of the probe hook indicates a horizontal tear at the posterior horn of the medial meniscus; d: the tear is sutured with the Fast-fix360 meniscus suture system under femoral condylar pushing technique, e, f: the meniscus is stable after suturing.

Under general anesthesia, a conventional anterolateral incision of about 4 mm near the patellar tendon was taken in the supine position. Each structure in the joint was explored after placing the endoscope successively, with the anterolateral approach as the observation channel. Under knee flexion and 20° eversion, the long needle was punctured from the medial patellar tendon to explore the suitable angle for suturing. Then, the anteromedial approach was established, with length of about 4-mm. Exposure enhancement technique was achieved by puncturing a 20-ml empty needle (With an inner diameter of 0.8mm and a length of 3cm) near the intersection of the medial joint line and the posterior bundle of the medial collateral ligament to release the posterior bundle of the medial collateral ligament, while valgus stress was applied until satisfactory gap. Firstly, the torn medial meniscus was repaired with a arthroscopic forceps, radical and irregular tears in the white area and red-white junction that were difficult to heal were removed, and those in the red area were retained. The injury of the posterior root of the medial meniscus was directly sutured using the Fast-fix360 system (Smith & Nephew, USA). The posterior horn of the medial meniscus obstructed the puncture of suture needle due to the occlusion of the medial condyle of the femur. Therefore, the femoral condyle pushing technique was performed by inserting the shaped metal channel for meniscus suture (Smith & Nephew, USA) into the medial approach and into the upper surface of the posterior horn of the medial meniscus. After the channel was overturned 180°, the groove surface pushed the meniscus towards the tibial plateau and far away from the cephalic side, with the medial condyle of the femur as the fulcrum. The suture needle of the Fast-fix360 system was inserted along the channel, accompanied by needle out and needle pulling. The metal channel was not pulled out until the suture was completed. The needle spacing was about 5 mm. Afterwards, the involution and stability of the meniscus were probed again, revealing satisfactory results. After the drainage tube was placed, the intra-articular fluid was sucked and then the skin incision was fully sutured. The surgical area was covered with sterile gauze, and the affected limb was dressed with pressure elastic bandage. The surgery was finished. The typical cases are shown in Fig.1.

### Postoperative treatment

Postoperatively, antibiotics were prophylactically used for 48 hour, and the suture was removed 10 days later. The patients were treated with protective external fixation with a knee brace and no weight bearing for four weeks, and followed up for six weeks, 12 weeks, six months and 12 months. The knee joint was allowed to move actively and passively 0-90° two weeks later, 120° four weeks later, and full range eight weeks later. Sports training was carried out three months after surgery.

### Evaluation method and statistical processing

The improvement in knee pain and range of activity were recorded, and the efficacy was determined according to the Lysholm scoring system.^9^ The data were expressed as mean ± standard deviation (x ± s), and statistically processed using SPSS18.0. The scores were statistically analyzed by the paired t-test. *P* < 0.05 was considered as statistically significant.

## RESULTS

All the 52 patients were followed up for 3-18 months after surgery, with an average of 12.5 ± 7.3 months. The pain and activity of all the patients were significantly improved compared with those before surgery. The average range of motion of the affected knee was 114.2° ± 10.7° before surgery and 131.5° ± 17.3° after surgery. Lysholm score was 65.9 ± 9.3 before surgery and 85.3 ± 7.1 after surgery. The pain and range of activity of the knee in all patients were significantly improved compared with those before surgery, and Lysholm score showed statistically significant (*P* < 0.05). No complications such as intra-articular or superficial infection and deep vein thrombosis of the lower extremity occurred in all patients.

## DISCUSSION

Meniscal injury is the most common knee injury in military and sports.^10^ Meniscal injury mostly occurs when the knee rotates while flexing, resulting in compression of the knee. Lateral meniscus injury is more common in acute injuries, with or without cruciate ligament injury.^11^-^13^ The injury of the medial meniscus, especially the injury of the posterior horn of the medial meniscus. It’s common in older people, but it’s also common in younger patients. At this time, the meniscus has degenerative changes at different degrees. Therefore, most injuries of the posterior horn of the medial meniscus occur on the basis of degenerative injuries.^14^-^17^ The diagnosis depends on MRI examination and sports medicine examination to a large extent, and it is often missed in primary hospitals. Conservative treatment is always the first treatment. Although there is no problem with the conservative treatment, the patients always have no obvious improvement in knee pain, or recurrence. It is suggested that MRI examination (1.5 T or above) should be performed in the outpatients aged 35-55 years with knee pain and no obvious intra-articular or valgus deformity, so as to make a definite diagnosis as early as possible, and arthroscopic surgery should be conducted if necessary. If the conditions permit (the position of meniscus tear, the patients’ economic conditions, etc.), the meniscus should be sutured as early as possible to avoid complete meniscectomy, which is of great significance to maintain the stability of the knee, relieve the pain, restore the range of activity and reduce the mobidity.

The torn meniscus can be divided into longitudinal tear, horizontal tear, radial tear and complex tear according to its shape. About 10% meniscal injuries are caused by the horizontal tear located in the red area or red-white junction of the meniscus. After resection of the white area and partial red-white junction, it is a potential position and shape of tear for suturing. In this study, Fast-fix360 suture system was used for the intraoperatively confirmed horizontal tear of the posterior horn of the medial meniscus. This system is specially designed for the suture of the posterior horn of the meniscus or the body, and can also be used for vertical mattress suture. The upper and lower part of the meniscus will bind and compress the tear. When operating the Fast-fix system, the action should be gentle. If the force is too strong or too fast or too slow, the suture anchor may be pulled out, leading to failure in suture. Through clinical research, some foreign investigators believe that the vertical mattress suture is more reliable than horizontal mattress suture or other suture methods in meniscus suture, and even vertical combined with horizontal mattress suture can be adopt. The authors believe that the horizontal tear of the posterior horn of the medial meniscus can be sutured by inserting the needle in the vertical direction, but embracing suture or binding suture is suitable. On the one hand, after repairing the posterior horn of the medial meniscus, the residual is small. On the other hand, this repair method belongs to “salvage” repair. In horizontal or vertical mattress suture, the arc of suture needle is difficult to penetrate the whole meniscus, thus the suture is not practical. It is difficult for the suture needle to enter into the upper surface of the posterior horn of the medial meniscus even in encircling suture or binding suture due to the block of the curved cartilage surface of the medial condyle of the femur. Therefore, the combined application of the two technologies was put forward here. Among them, the exposure enhancement technique is commonly used in arthroscopy to expose the medial space, which is always used in the surgery without anterior cruciate ligament injury or medial collateral ligament injury.

The femoral condyle pushing technique is an innovative technique, similar to the reduction technique with folding manipulation for fracture end in orthopaedic trauma. By this technique, the meniscus surface to be inserted is pushed and pressed down with a certain device using the lever principle, so as to complete the suture. To ensure the healing of the horizontal tear of the meniscus, the needle spacing should not be too large. In this study, needle spacing of about 5 mm was selected, and generally 2-3 needles could meet the requirements for repair. Our 52 patients were all treated with the combination of the two techniques, with satisfactory efficacy and no additional tears. The results of this study showed that the knee pain and range of motion of all patients were significantly improved compared with that before surgery. The results were similar to those of Mochizuki Y et al.^18^ and Shi WJ et al.^19^ Ahn JH et al., in a case study, showed an improvement in mean knee score after arthroscopic anterior cruciate ligament reconstruction with total internal hook sutures.^20^ In this study, patients’ Lysholm score after surgery was significantly improved compared with that before surgery, and the difference was statistically significant, which was similar to the results of previous studies.

Attention should be paid to in the application of these techniques as follows: (1) The indications should be strictly grasped. The meniscus which is not suitable for suturing (determined by the shape of tear, texture, etc.) should be removed decisively. (2) Arthroscopic surgery has relatively high requirements for instruments. It is better to have a shaped metal meniscus suture channel, which can be replaced with metal instruments with width of 3-4 mm, thickness of 0.5-1 mm and length of 10-15 cm. (3) At present, the cost of meniscus suture device in China is relatively high, and there is a certain risk of nonunion after suture. If necessary, the meniscus needs to be removed by secondary surgery, and the patients should be fully communicated with before surgery.

### Limitations of this study

This was a retrospective study with limited data integrity and homogeneity. It is necessary to further design a randomized controlled trial to verify the observations in this study. In addition, there are few studies on exposure enhancement technique and femoral condyle pushing technique in previous studies, which proves the innovation of this study, but also makes the discussion of this study insufficient.

## CONCLUSION

This study demonstrates that the exposure enhancement technique combined with femoral condyle pushing technique has satisfactory efficacy in repairing the posterior horn of the medial meniscus, and can improve the pain and activity of the knee and enhance the stability of the residual meniscus. Therefore, it can be promoted and applied in qualified medical institutions. Long-term follow-up and evaluation of this surgery are being further investigated and summarized.

### Authors’ Contributions:

**XL & DX:** designed this study and prepared this manuscript, are responsible and accountable for the accuracy and integrity of the work.

**TH & CX:** Collected and analyzed clinical data.

**YZ:** Significantly revised this manuscript.
